# Supplementary material for: Pyruvate Dehydrogenase Kinase 1 inhibition mediated oxidative phosphorylation enhancement in cartilage promotes osteoarthritis progression
Source: BMC Musculoskelet Disord. 2023 Jul 20;24:597. doi: 10.1186/s12891-023-06585-6 (PMC10357736; doi:10.1186/s12891-023-06585-6)
Supplement: Supplementary file 1 — Additional file 1. [file 12891_2023_6585_MOESM1_ESM.zip › BP.Gsea.1653623667859/GOBP_SOMATIC_DIVERSIFICATION_OF_IMMUNE_RECEPTORS_VIA_SOMATIC_MUTATION.html]

Details for gene set GOBP\_SOMATIC\_DIVERSIFICATION\_OF\_IMMUNE\_RECEPTORS\_VIA\_SOMATIC\_MUTATION[GSEA]

|  || Dataset | 55457\_collapsed\_to\_symbols.cls.cls #OA\_versus\_normal.cls.cls #OA\_versus\_normal\_repos |
| Phenotype | cls.cls#OA\_versus\_normal\_repos |
| Upregulated in class | OA |
| GeneSet | GOBP\_SOMATIC\_DIVERSIFICATION\_OF\_IMMUNE\_RECEPTORS\_VIA\_SOMATIC\_MUTATION |
| Enrichment Score (ES) | 0.5732182 |
| Normalized Enrichment Score (NES) | 1.6908145 |
| Nominal p-value | 0.014285714 |
| FDR q-value | 0.16844232 |
| FWER p-Value | 1.0 |
Table: GSEA Results Summary

  

Fig 1: Enrichment plot: GOBP\_SOMATIC\_DIVERSIFICATION\_OF\_IMMUNE\_RECEPTORS\_VIA\_SOMATIC\_MUTATION      
 Profile of the Running ES Score & Positions of GeneSet Members on the Rank Ordered List

  

| SYMBOL | TITLE | RANK IN GENE LIST | RANK METRIC SCORE | RUNNING ES | CORE ENRICHMENT || 1 | POLB | DNA polymerase beta [Source:HGNC Symbol;Acc:HGNC:9174] | 93 | 0.923 | 0.2065 | Yes |
| 2 | MLH1 | mutL homolog 1 [Source:HGNC Symbol;Acc:HGNC:7127] | 497 | 0.660 | 0.3281 | Yes |
| 3 | MSH2 | mutS homolog 2 [Source:HGNC Symbol;Acc:HGNC:7325] | 739 | 0.584 | 0.4446 | Yes |
| 4 | PMS2P1 | "PMS1 homolog 2, mismatch repair system component pseudogene 1 [Source:HGNC Symbol;Acc:HGNC:9123]" | 917 | 0.537 | 0.5553 | Yes |
| 5 | AICDA | activation induced cytidine deaminase [Source:HGNC Symbol;Acc:HGNC:13203] | 1825 | 0.381 | 0.5732 | Yes |
| 6 | POLQ | DNA polymerase theta [Source:HGNC Symbol;Acc:HGNC:9186] | 4293 | 0.129 | 0.4115 | No |
| 7 | PMS2P5 | "PMS1 homolog 2, mismatch repair system component pseudogene 5 [Source:HGNC Symbol;Acc:HGNC:9130]" | 4623 | 0.103 | 0.4098 | No |
| 8 | SAMHD1 | SAM and HD domain containing deoxynucleoside triphosphate triphosphohydrolase 1 [Source:HGNC Symbol;Acc:HGNC:15925] | 5790 | 0.009 | 0.3214 | No |
| 9 | UNG | uracil DNA glycosylase [Source:HGNC Symbol;Acc:HGNC:12572] | 6078 | -0.011 | 0.3015 | No |
| 10 | POLL | DNA polymerase lambda [Source:HGNC Symbol;Acc:HGNC:9184] | 6214 | -0.022 | 0.2961 | No |
| 11 | MCM3AP | minichromosome maintenance complex component 3 associated protein [Source:HGNC Symbol;Acc:HGNC:6946] | 8077 | -0.161 | 0.1889 | No |
| 12 | EXO1 | exonuclease 1 [Source:HGNC Symbol;Acc:HGNC:3511] | 8088 | -0.162 | 0.2258 | No |
| 13 | PMS2 | "PMS1 homolog 2, mismatch repair system component [Source:HGNC Symbol;Acc:HGNC:9122]" | 8172 | -0.169 | 0.2584 | No |
| 14 | MSH6 | mutS homolog 6 [Source:HGNC Symbol;Acc:HGNC:7329] | 8846 | -0.221 | 0.2573 | No |
| 15 | ADAR | adenosine deaminase RNA specific [Source:HGNC Symbol;Acc:HGNC:225] | 9195 | -0.247 | 0.2875 | No |
Table: GSEA details [plain text format]

  

Fig 2: GOBP\_SOMATIC\_DIVERSIFICATION\_OF\_IMMUNE\_RECEPTORS\_VIA\_SOMATIC\_MUTATION      
 Blue-Pink O' Gram in the Space of the Analyzed GeneSet

  

Fig 3: GOBP\_SOMATIC\_DIVERSIFICATION\_OF\_IMMUNE\_RECEPTORS\_VIA\_SOMATIC\_MUTATION: Random ES distribution      
 Gene set null distribution of ES for **GOBP\_SOMATIC\_DIVERSIFICATION\_OF\_IMMUNE\_RECEPTORS\_VIA\_SOMATIC\_MUTATION**
